# Nocardia farcinica Brain Abscess in an Immunocompetent Host With Pulmonary Alveolar Proteinosis: A Case Report and Review of the Literature

**DOI:** 10.7759/cureus.11494

**Published:** 2020-11-15

**Authors:** Sarah E Grond, Alyssa Schaller, Alexandra Kalinowski, Kimberly A Tyler, Pinky Jha

**Affiliations:** 1 Internal Medicine, Medical College of Wisconsin, Wauwatosa, USA; 2 Pharmacy, Concordia University of Wisconsin, Mequon, USA

**Keywords:** nocardia species, pulmonary alveolar proteinosis, disseminated nocardiosis, brain abscess

## Abstract

A 55-year-old immunocompetent male presented with new-onset seizures and acute respiratory failure requiring intubation and a stay in the medical intensive care unit. Magnetic Resonance Imaging (MRI) of the brain revealed ring-enhancing lesions, and Computed Tomography (CT) chest showed ground-glass opacity. The patient underwent craniotomy and bronchoscopy, followed by culture of the purulent aspirate from lesions in the brain and bronchoalveolar lavage (BAL). After extensive infectious workup, the patient was diagnosed with a *Nocardia farcinica* brain abscess plus underlying pulmonary alveolar proteinosis (PAP). Based on a recommendation from an infectious disease expert, the patient was treated with trimethoprim-sulfamethoxazole (TMP/SMX).

This case highlights the importance of understanding that, though rare, infections such as nocardiosis can present in immunocompetent patients and cause severe morbidity.

## Introduction

*Nocardia* species are a group of obligate aerobic soil saprophytes that belong to the order Actinomycetales. They are weakly acid-fast bacilli that form branching filaments, structures that resemble the hyphae formed by fungi, and can often be deceiving on culture [1,2]. Over 80 *Nocardia* species have been identified, and roughly half of those organisms are clinically significant. *Nocardia asteroides *has historically been implicated most often in human disease; however, it is now divided into six separate species and renamed *N. asteroides* complex, of which *N. abscessus, N. nova*, and *N. farcinica* are some of the most clinically relevant human pathogens [3]. *N. farcinica* has been shown to have greater virulence than other Nocardia species, and it is commonly implicated in systemic infection [3]. *N. brasilensis* is another common human pathogen that most often causes cutaneous infections [3].

Every year about 500 to 1000 new cases of *Nocardia* infections are reported, most often in patients who have preexisting immune system compromise [4,5]. The average age of infection onset is 40, and infections typically occur more often in males than females. *Nocardia* species are opportunistic pathogens; therefore, immunocompromised patients with conditions such as diabetes, cancer, lupus, and inflammatory bowel disease (IBD), are more at risk for infection [5]. *Nocardia* species can be found in water, soil, and decaying plants, as well as in dust and swimming pools, and transmission occurs via inhalation of the microorganisms or through skin inoculation through open wounds [1,2].

Here we report a case of a *Nocardia farcinica* brain abscess in an immunocompetent host with pulmonary alveolar proteinosis (PAP).

## Case presentation

**Figure 1:**
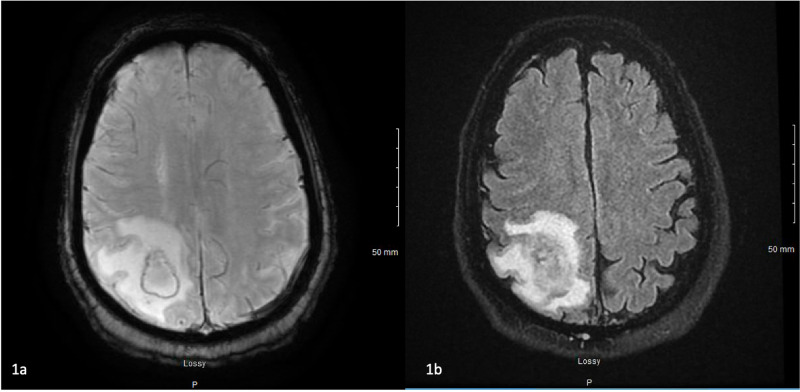
Initial MRI head showing 3.1 x 2.3 cm ring-enhancing lesion in the right parietal lobe Figure 1a: axial susceptibility-weighted imaging (SWI), Figure 1b: axial fluid-attenuated inversion-recovery (FLAIR) fat-suppressed (FS) imaging

A 51-year-old African American male with a medical history of psychosis, hypertension, and alcohol abuse presented with new-onset seizures, manifesting as repetitive movements of the left upper extremity. On review of systems, the patient also endorsed recent subjective fevers, cough, shortness of breath, and a 40- to 50-pound weight loss over the last four months. Neurology was consulted for the status epilepticus, and workup included electroencephalogram (EEG), computed tomography (CT), and magnetic resonance imaging (MRI). Brain imaging revealed a 3.1 x 2.3 cm mass in the right parietal lobe with surrounding edema, described as a ring-enhancing lesion (Figure 1). Significant laboratory findings included respiratory acidosis, lactic acid of 15.4 mmol/L, elevated ferritin of 833 ng/mL, and elevated lactate dehydrogenase (LDH) of 390 unit/L (Table 1). Subsequent chest x-ray (CXR) revealed a diffuse interstitial prominence. Chest imaging, along with respiratory symptoms, raised suspicion for COVID-19; however, viral swabs were negative.

**Table 1:** Initial laboratory workup, including reference ranges EGFR = estimated glomerular filtration rate, AST = aspartate aminotransferase, ALT = alanine aminotransferase, LDH = lactate dehydrogenase, PT = prothrombin time, INR = International Normalized Ratio, PTT = partial thromboplastin time, SGPT = serum glutamic-pyruvic transaminase, SGOT = serum glutamic-oxaloacetic transaminase, NAAT = nucleic acid amplification test, AU = arbitrary unit, AB = antibody, AG = antigen, ANA = antinuclear antibody, AMA = American Medical Association, ID = immunodiffusion, CF = complement fixation

Test Name	Test Results (Reference Range)
White Blood Cell Count	9.8 (3.9-11.2 x 10e3/uL)
Red Blood Cell Count	4.9 (4.4-5.9 x 10e6/uL)
Hemoglobin	16.1 (13.7-17.5 g/dL)
Hematocrit	50 (40-51%)
Mean Corpuscular Volume	103 (79-98 fL)
Mean Corpuscular Hemoglobin	33.1 (25.7-32.2 pg)
Mean Corpuscular Hemoglobin Concentration	32 (32-36 g/dL)
Red Cell Distribution Width	12.5 (11-14.9%)
Platelet Count	287 (165-366 x 10e3/uL)
Mean Platelet Count	9.4 (9.0-11.8 fL)
Automated Nucleated Red Blood Cells	0 (0-0%)
Blood Urea Nitrogen	6 (6-23 mg/dL)
Sodium	143 (136-145 mmol/L)
Potassium	4.8 (3.4-5.1 mmol/L)
Chloride	99 (96-105 mmol/L)
Anion Gap	37 (10-18 mmol/L)
Bicarbonate	7 (22-29 mmol/L)
Glucose	194 (65-99 mg/dL)
Creatinine	1.14 (0.70-1.30 mg/dL)
EGFR	>60 mL/min/1.73 sqm
Albumin	4.9 (3.8-5.0 g/dL)
Total Protein	9.2 (6.1-8.2 g/dL)
Calcium	10.0 (8.6-10.2 mg/dL)
Alkaline Phosphatase	111 (40-129 unit/L)
AST	103 (13-44 unit/L)
Bilirubin Total	0.9 (0.2-1.2 mg/dL)
ALT	37 (8-66 unit/L)
Magnesium	2.2 (1.6-2.6 mg/dL)
Phosphorus	5.6 (2.5-4.5 mg/dL)
C-Reactive Protein	0.90 (0.00-0.50 mg/dL)
D-Dimer Quantitative	1.31 (<=0.69)
Ferritin	833.0 (30.0-400.0 ng/mL)
LDH	390 (135-225 unit/L)
Procalcitonin	0.10 (<=0.08 ng/mL)
Arterial Blood Gas	7.19 (7.35-7.45)
PT/INR	PT- 10.2 (9.5-11.2 seconds), INR - 1.0
PTT	23.4 (23.0-30.0 seconds)
Manual Differential	Segmented neutrophils - 26 (43-74%), Lymphocytes - 62 (17-46%), Monocytes - 11 (4-13%), Eosinophils - 0 (0-6%), Basophils - 1 (0-1%), RBC Morphology - normal, Absolute neutrophil - 2.55 (1.90-7.80 x 10e3/uL), Absolute lymphocyte - 6.08 (0.90-3.20 x 10e3/uL), Absolute monocytes - 1.08 (0.26-0.86 x 10e3/uL), Absolute basophils - 0.10 (0.01-0.10 x 10e3/uL)
Blood Peripheral Path Review	Mild absolute lymphocytosis with reactive features.
Hepatic Function Panel	Albumin - 4.9 (3.8-5.0 g/dL), ALT/SGPT - 34 (8-66 unit/L), AST/SGOT - 106 (13-44 unit/L), Alkaline Phosphatase - 111 (40-129 unit/L), Total Protein - 9.3 (6.1-8.2 g/dL), Bilirubin Total - 0.9 (0.2-1.2 mg/dL), Direct Bilirubin - 0.2 (0.0-0.3 mg/dL)
Culture Blood	No Growth after 48 hours
Lactic Acid Reflex - Sepsis	15.4 (0.5-2.0 mmol/L)
Coronavirus COVID-19	Not detected
Influenza A and B Virus NAAT	Influenza A and B Virus NAAT - Not detected
Respiratory Extended Pathogen Panel	Adenovirus NAAT - Not detected, Coronavirus 229E - Not detected, Coronavirus HKU1 - Not detected, Coronavirus NL63 - Not detected, Coronavirus OC43 - Not detected, Human Metapneumovirus - Not detected, Human Rhinovirus NAAT - Not detected, Influenza A Virus NAAT - Not detected, Influenza B Virus NAAT - Not detected, Parainfluenza 1 Virus NAAT - Not detected, Parainfluenza 2 Virus NAAT - Not detected, Parainfluenza 3 Virus NAAT - Not detected, Parainfluenza 4 Virus NAAT - Not detected, Respiratory Syncytial Virus NAAT - Not detected, B. Parapertussis NAAT - Not detected, B. Pertussis NAAT - Not detected, Chlamydia pneumoniae NAAT - Not detected, Mycoplasma pneumoniae NAAT - Not detected
Ammonia	36 (16-60 umol/L)
HIV ½ AB/AG, 4th Generation	Nonreactive
Coccidioides Panel	Coccidioides ID Antibody - None detected, Coccidioides CF Antibody - <1:2 (<1:2)
Toxoplasma IgG+IgM Panel	Toxoplasmosis IgG Screen <0.2 (international units/mL), Toxoplasma IgM Antibody <3.0 (<=7.9 AU/mL)
Cryptococcal Antigen Serum	Not detected
ANA Reflex to Comprehensive Profile	Nuclear AB Screen < 0.2 (0.0-0.9 Al)
Hepatitis Panel AMA Acute	Hepatitis IgM Antibody - Nonreactive, Hepatitis B Core IgM Antibody - Nonreactive, Hepatitis B Surface Antigen - Nonreactive, Hepatitis C Antibody - Nonreactive
9 Drug Panel Urine	Ethanol - <0.010 (<= 0.010 g/dL), Amphet/Methamphetamine - Negative, Barbiturates - Negative, Benzodiazepine - Negative, Cannabinoid - Negative, Cocaine - Negative, Methadone - Negative, Opiates - Negative, Oxycodone - Negative
Histoplasma Antigen Urine	None Detected
Blastomyces Antigen Urine	None Detected
Aspergillus Galactomannan Antigen	Negative
Atypical Pneumonia NAAT	Mycoplasma pneumoniae NAAT - Negative, Chlamydia pneumoniae NAAT - Negative, Legionella pneumoniae NAAT – Negative

Seizure control was initially attempted with levetiracetam, divalproex sodium, and fosphenytoin but required escalation to lacosamide and phenobarbital. On day one of hospitalization, the patient had worsening lethargy and mental status, so the patient was intubated. A decision was made to proceed with needle biopsy of the brain abscess, which revealed purulent fluid that grew branching, gram-positive rods, which were later confirmed to be *Nocardia farcinica*. An infectious disease expert was consulted, and the patient was started on cefepime, trimethoprim-sulfamethoxazole (TMP-SMX), and metronidazole for coverage of *Nocardia, Blastomycosis, *and* Cryptococcus* species. 

**Figure 2:**
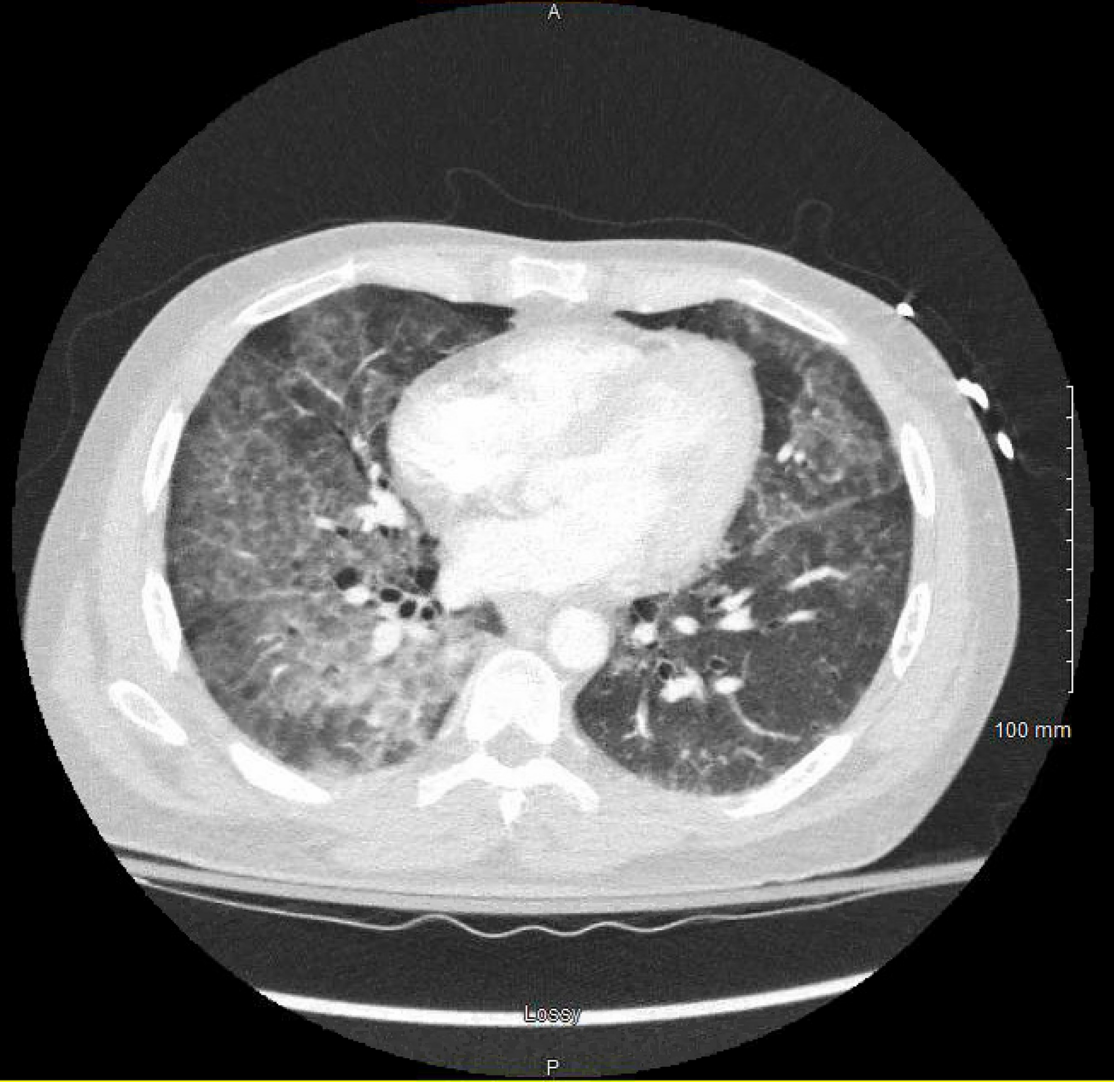
Chest CT showing ground-glass opacities

Bronchoscopy with bronchoalveolar lavage (BAL) was performed due to the observation of ground-glass opacities on CT (Figure 2). Secretions were thick and purulent with a "milky" appearance, and a gram stain resulted in rare gram-negative rods and gram-positive cocci. Periodic acid-Schiff (PAS) stain was positive. The findings were consistent with PAP. Given the diagnosis of *Nocardia farcinica* brain abscess, additional infectious workup was ordered, and no other infectious pathogens were identified, including negative human immunodeficiency virus (HIV) test (Table 1). Due to the suspected PAP in the setting of acute hypoxemic respiratory failure, a right whole lung lavage was performed. Post-operatively the patient developed intermittent fevers, and linezolid and moxifloxacin were added for additional coverage. Blood cultures were sent and had no growth. Given the patient’s clinical improvement, antibiotics were narrowed back to TMP-SMX. 

Surveillance MRI on hospital day 13 showed an increase in the previously drained right parietal abscess. Antibiotics were escalated to oral TMP-SMX, intravenous (IV) metronidazole, ceftriaxone, and vancomycin. A repeat craniotomy was performed for drainage, and *Nocardia farcinica* continued to be the only pathogen present. Antibiotics were narrowed back down to TMP-SMX. At this point, the patient experienced another seizure, requiring adjustments of his levetiracetam and phenobarbital. The patient became febrile once again, and antibiotics were switched to IV TMP-SMX, linezolid 600 mg every 12 hours, and moxifloxacin 400 mg four times a day. Soon after, the patient was noted to have increased secretions and became increasingly lethargic and unarousable, requiring intubation again. The left whole lung lavage was scheduled for suspected PAP and proceeded as planned. The patient was successfully extubated soon after, and antibiotics were tapered back down to TMP-SMX. 

By week three of hospitalization, a repeat MRI showed improvement of the cerebral abscess and edema. The patient continued to improve clinically and was discharged to neurologic rehabilitation on hospital day 34 with plans for weekly basic metabolic panels (BMPs) and two double-strength TMP-SMX three times daily for one year, in addition to continuing dexamethasone 4 mg twice a day, clobazam 10 mg twice a day, lacosamide 200 mg twice a day, levetiracetam 1250 mg twice a day, NaCl 2 g three times daily. 

Six weeks post-discharge, follow-up CT showed worsening bilateral ground-glass opacities with interlobular septal thickening and new consolidated, bilateral opacities. The patient was admitted for scheduled bronchoalveolar lavages, which proceeded as planned without complication. 

## Discussion

*Nocardia* species are a group of obligate aerobic soil saprophytes that most often cause pulmonary, central nervous system, and cutaneous infection in immunocompromised hosts [1]. Nocardiosis, though rare, can cause severe disease and widespread disseminated infection. Though *Nocardia* species are commonly found in soil, infection rates are low. Additionally, the majority of infections are caused by inhalation, and therefore pulmonary involvement is the most likely initial presentation [4]. Other typical manifestations include brain abscesses and skin/soft tissue infection. The majority of *Nocardia* infections are diagnosed in immunocompromised hosts; however, at least 35% of patients are immunocompetent at the time of diagnosis [1].

Immunocompetence is defined as having an appropriate immune response following exposure to a foreign antigen. Since pulmonary alveolar proteinosis (PAP) is not considered an immunodeficiency, the patient was determined to be immunocompetent in absence of immunodeficiency. Here we report a case of a *Nocardia farcinica *brain abscess and PAP in an otherwise immunocompetent host.

We conducted an extensive literature search of* Nocardia farcinica* infections in immunocompetent hosts. Eighteen previous cases of* N. farcinica* in immunocompetent hosts were found, and those between 2010 and 2020 can be seen in Table 2. We did not find any case reports that highlighted *Nocardia farcinica *infections in patients with PAP.

**Table 2:** Literature review of Nocardia farcinica infections in immunocompetent hosts between 2010-2020 HTN = hypertension, GERD= gastroesophageal reflux disease, CAD = coronary artery disease, COPD=Chronic Obstructive Pulmonary Disease

Authors, Year of Study	Nocardia dissemination	Immunocompetent?	Other comorbidities
Vuotto et al., 2011 [6]	Iliac artery stent infection	Yes	Iliac artery stenosis
Budzik et al., 2012 [7]	Pneumonia and synovitis	Yes	One time steroid injection in knee
Kim et al., 2014 [8]	Brain abscess	Yes	HTN
Kim et al., 2016 [9]	Pneumonia, mediastinitis	Yes	HTN
Boamah et al., 2016 [10]	Pneumonia, brain abscess	Yes	Smoking, GERD
Pascual-Gallego et al., 2016 [11]	Brain abscess (cerebellum)	Yes	Olfactory groove meningioma 11 years earlier
Jackson et al., 2017 [12]	Pneumonia, adrenal abscess, brain abscess	Yes	CAD, smoking
Chaudhari et al., 2017 [13]	Brain abscess	Yes	Diabetes (well-controlled)
Holmes et al., 2018 [14]	Brain abscess	Yes	COPD, HTN
Faircloth and Troy, 2019 [15]	Pericardial effusion and pericarditis	Yes	Alcohol consumption
Wang et al., 2019 [16]	Orbital infection	Yes	None
Moniuszko-Malinowska et al., 2020 [17]	Chronic meningitis	Yes	None

The clinical presentation of *Nocardia* infections varies depending on organ system involvement; however, the symptoms tend to be nonspecific and require further workup. The most common infections are cutaneous, pulmonary, and neurological; however, kidney, spleen, liver, bone, and joint infections may also occur [1]. Patients may present with fever, weight loss, night sweats, cough, or chest pain, but they can also present with headaches and seizures if there is neurological involvement [4]. Patients with cutaneous infections will often present with erythema, nodules, or ulcers [1]. Prior to diagnosis, a full patient history should be obtained along with proper imaging. This imaging should include CT, chest X-ray, and MRI, along with appropriate neurological imaging and assessment to rule out any central nervous system (CNS) involvement. Specimen collection can include biopsy, bronchoscopy, aspiration, as well as sputum collection, and a Gram stain and modified acid-fast stain should be used for culturing [5].

About 39% of patients present with pulmonary *Nocardia* infection; however, approximately 32% of those cases disseminate to other organ systems [1]. Patients with pulmonary involvement often present with necrotizing pneumonia, which can lead to pleural effusions, empyemas, as well as pericarditis. Over 44% of all cases have CNS involvement, which is most often secondary to pulmonary infection [1]. Patients who are immunocompromised may have asymptomatic abscesses that exhibit a latency period for up to three years; however, when these patients present with symptoms, the infection is typically disseminated and more life-threatening. Additionally, primary cutaneous and soft tissue nocardiosis can occur as a result of direct inoculation of the skin [18]. There are three types of cutaneous infections, including cellulitis, actinomycetomas, and lymphocutaneous disease. Actinomycetomas involve bone and subcutaneous tissue and typically have a late-onset after infection, whereas lymphocutaneous disease results in pus, drainage, and crusting of lesions and can lead to lymphatic abscesses [1,18].

There are currently no formal treatment guidelines for managing *Nocardia* infections, which highlights the need for future research and the development of treatment protocols based on randomized controlled trials. Treatment of *Nocardia* infections is highly individualized, and susceptibility testing is key as antimicrobial resistance is common among different *Nocardia* species [19]. Monotherapy is typically sufficient for immunocompetent hosts with systemic or cutaneous infection; however, in immunocompromised patients or in patients with pulmonary or disseminated infection, double or triple therapy is often warranted [5]. The duration of therapy varies widely, and individual patient factors must be considered. Immunocompetent patients with cutaneous infection may need only a course of one to three months of antibiotic therapy, whereas uncomplicated pulmonary infections may require treatment for less than six months. In immunocompromised patients, patients with disseminated infection, or patients with CNS involvement, a minimum of six months of antimicrobial therapy is typically used, and patients are monitored thereafter for infection recurrence [5]. In addition, surgical management may be necessary, especially in those with severe disease and failure of antimicrobial therapy [1,20].

Various drug susceptibility analyses have been performed. Sulfonamides, aminoglycosides, beta-lactam/beta-lactamase inhibitors, fluoroquinolones, macrolides, and tetracyclines have all been explored as treatment options for *Nocardia* infections. Sulfonamide antimicrobials, such as TMP-SMX, are the treatment agents of choice. *Nocardia farcinica* has been noted to have increased antibiotic resistance compared to other strains, with resistance to beta-lactams and aminoglycosides but susceptibility to amoxicillin-clavulanic acid, amikacin, moxifloxacin, linezolid, and TMP-SMX and variable susceptibility toward imipenem and ciprofloxacin [19]. Before antibiotic susceptibility tests return, empiric therapy of TMP-SMX, amikacin, and either ceftriaxone or imipenem is usually initiated [3].

To the best of our knowledge, this case report is the first case of a *Nocardia farcinica* brain abscess in a patient with underlying PAP.

## Conclusions

In conclusion, we present a case of a *Nocardia farcinica *brain abscess in an immunocompetent patient with pulmonary alveolar proteinosis. With this case, we hope to increase awareness among practitioners about this challenging diagnosis, especially in immunocompetent hosts. Additionally, *Nocardia farcinica *is a rare and potentially fatal infection that is difficult to treat and often requires a multidisciplinary approach.
